# Mutation analysis of the *GSDME* gene in a Chinese family with non-syndromic hearing loss

**DOI:** 10.1371/journal.pone.0276233

**Published:** 2022-11-09

**Authors:** Peiliang Lei, Qingwen Zhu, Wenrong Dong, Siqi Zhang, Yanyan Sun, Xitong Du, Meng Geng, Yuan Jiang

**Affiliations:** 1 Department of Otolaryngology Head & Neck Surgery, The Second Hospital of Hebei Medical University, Shijiazhuang, Hebei, 050000, China; 2 Department of Otolaryngology Head & Neck Surgery, The Third Hospital of Shijiazhuang, Shijiazhuang, Hebei, 050011, China; University of Iowa, UNITED STATES

## Abstract

**Background:**

Hearing loss is considered one of the most common sensory nervous system defects, about 60% of which are caused by genetic factors. Mutations in the *GSDME* gene are responsible for post-lingual, progressive, autosomal dominant hearing loss. This study aimed to characterize the genetic mutations and clinical features of a Chinese *GSDME* family.

**Methods:**

After clinical evaluations, high-throughput DNA sequencing was conducted using DNA samples from this family. Sanger sequencing was performed to verify the suspected variants. A detailed genotype and phenotype analysis were carried out. Gene set enrichment analysis (GSEA) was performed to identify the signaling pathway associated with *GSDME* expression.

**Results:**

A known hotspot heterozygous splice-site variation (c.991-15_991_13delTTC) was identified and shown to segregate with the hearing loss phenotype in the family. This pathogenic splice-site variant results in skipping of exon 8. GSEA analysis identified changes in regulation of the cell cycle checkpoint, peroxisome, and amino acid metabolism signaling pathways.

**Conclusions:**

We identified a reported mutation in the *GSDME* gene. Our findings support the 3 bp deletion (c.991-15_991-13del) was a hotspot variation, and it emerged as an essential contributor to autosomal dominant progressive hearing loss in East Asians. *GSDME* gene is closely associated with a range of signaling pathways. These characterized findings may provide new evidence for pathogenesis.

## Introduction

Hearing loss is one of the most common clinically disabling diseases. According to the World Health Organization, more than 5% of the world’s population—466 million people—suffer from hearing loss of varying degrees [1]. Genetic defects, body aging, viral and bacterial infections, drug-induced ototoxicity, trauma, and exposure to noisy environments contribute to hearing loss, about 60% of which are caused by hereditary factors. Hereditary hearing loss can be divided into syndromic deafness and non-syndromic deafness, depending on whether other organ abnormalities accompany it. At present, 124 genes associated with hereditary non-syndromic hearing loss have been identified, including 51 genes for autosomal dominant hearing loss, 78 genes for autosomal recessive hearing loss, and 5 genes for X-linked inheritance(https://hereditaryhearingloss.org/). Autosomal dominant non-syndromic hearing loss (ADNSHL) accounts for approximately 20% of non-syndromic hereditary hearing loss [2].

Hereditary hearing loss exhibits a high degree of genetic heterogeneity. *GSDME*(MIM:600994) was mapped to chromosome 7p15 in 1995 and identified in 1998 in a Dutch family [3,4]. In later studies, *GSDME* had also been detected to play an essential role as an oncogene [5]. The presumed mechanism is that *GSDME* could be activated by Caspase-3 cleavage, leading to apoptosis and cytokinesis transition [6]. However, the biological function of *GSDME* and the upstream pathways need to be further investigated. To date, several mutations in *GSDME* have been reported to cause ADNSHL [7–14]. These reported mutations lead to the skipping of exon 8 at the mRNA level, resulting in a frameshift and producing a prematurely truncated GSDME protein. In this study, we present the identification of a *GSDME* mutation, c.991-15_991_13delTTC, in a Chinese family. The TTC deletion in the polypyrimidine tract of intron 7 also led to exon 8 skipping in mRNA level and subsequently to non-syndromic sensorineural hearing impairment.

## Methods

### Pedigree and clinical evaluation

**Fig 1:**
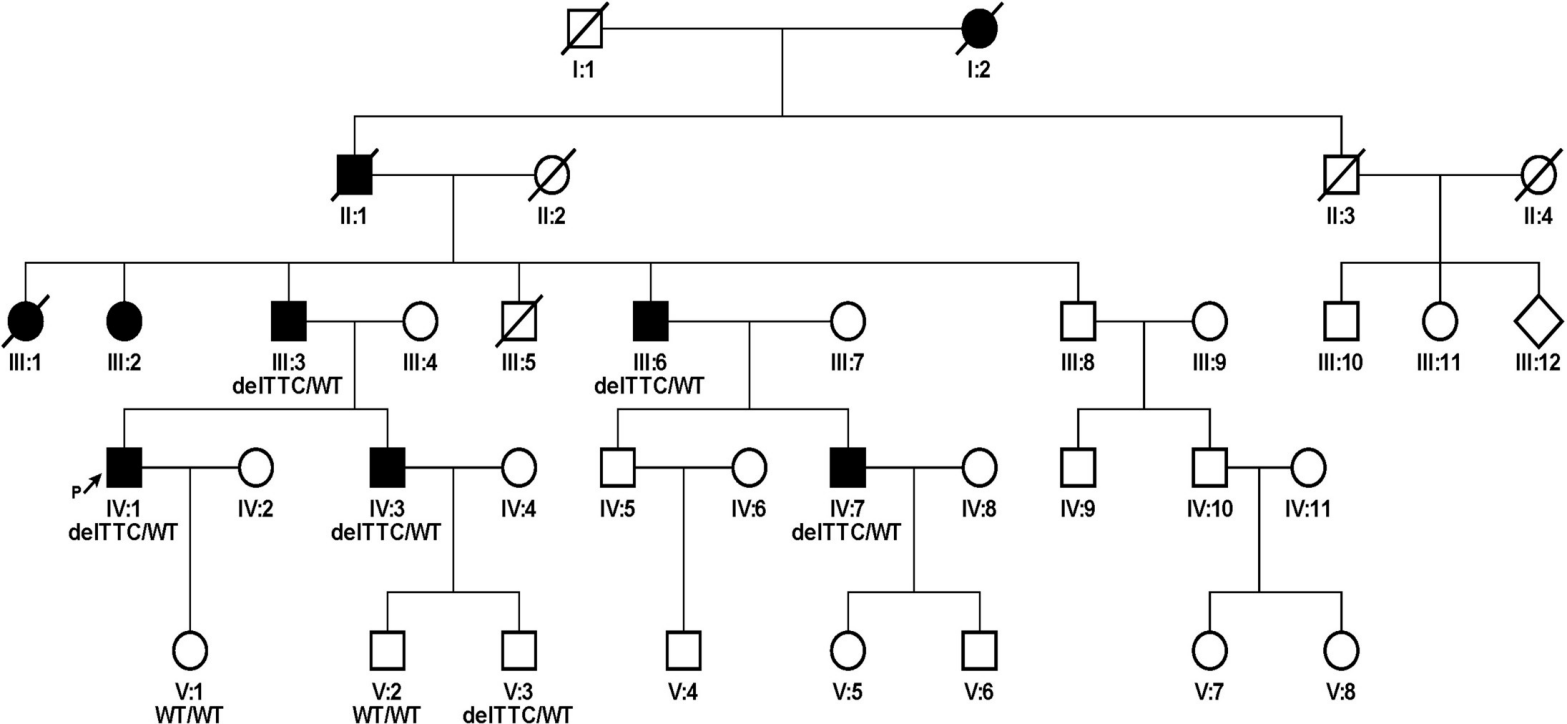
Pedigree of the family. ↗: Proband; Mutation analysis (III2,III:3, III:6, IV:1, IV:3, V:1, V:2, V:3); Pure-tone audiometry performed (III:2, III:3, III:4, III:6, IV:1, IV:2, IV:3, V:1, V:2, V:3).

All procedures were approved by the Ethics Reviewing Committee of the Second Hospital of Hebei Medical University. All subjects or their parents provided written informed consent to participate in the study. A five-generation hearing loss-affected family from Hebei Province was collected (Fig 1) in 2019. The following information was obtained from all participants through several interviews and completed questionnaires: Age at onset and triggers, subjective degree of hearing loss, presence of tinnitus and vertigo, the evolution of the hearing loss, history of head trauma, and medication with aminoglycosides, noise exposure, pathological changes in the ear and other relevant clinical manifestations.

All subjects received an otolaryngological clinical examination, including otoscopy, physical examination, pure tone audiometry (frequencies from 0.125 to 8kHz), acoustic conductance resistance, and computed tomography scans of the temporal bone. Hearing curves were drawn to determine the degree and type of hearing loss. The diagnosis of sensorineural hearing loss was made according to the WHO criteria.

A literature search using the PubMed database retrieved published audiometric data for non-syndromic deafness families segregating the *GSDME* mutation. From these literature data, patient profiles containing age information and pure-tone audiograms (binaural symmetry) were selected. Only records from the first visit of each selected patient were included. In the collected dataset, there were 6 records from this study, 13 records for the delTTC pathogenic variant and 44 records for other pathogenic variants in *GSDME*. After calculating the binaural average air conduction thresholds from 0.25 to 8 kHz, we performed cross-sectional and longitudinal analyses with linear regression to evaluate the progression of hearing impairment for each frequency. Progression is considered significant if the 95% confidence interval of the slope does not include zero, expressed as annual threshold deterioration (ATD; dB per year). We analyzed the progression rates for each frequency in the three groups: this study, delTTC variant, and other variants. As previously described [15,16], age-related typical audiograms (ARTA) were constructed. Regression data were used to derive the ARTA and to predict the expected hearing threshold at fixed ages (10–70 years). Statistical analyses were performed, and graphs were generated with GraphPad Prism 8.3.0.

### Targeted genomic capture and variation analysis

According to the manufacturer’s guidelines, DNA was extracted from peripheral blood samples from all subjects using a blood DNA extraction kit (TIANGEN, Beijing, China). The proband was examined using a gene panel containing 415 genes related to deafness. The amplified DNA was captured using the GenCap deafness capture kit (MyGenostics, Beijing, China). We selected Exons, and the flanking 50 bp for capture and NGS sequencing was performed on an Illumina HiSeq2000 (Illumina, San Diego, CA, USA). The BWA software mapped the clean reads to the GRCh37/hg19 human reference genome. Variants were annotated by ANNOVAR and compared with multiple databases. Potential pathogenic variants were filtered with a minimum allele frequency threshold ≤0:001 for dominant inheritance. The Human Gene Mutation Database (HGMD) and the ClinVar database were searched for reported mutation, and reported benign variants were excluded. The pathogenicity was evaluated according to the American College of Medical Genetics and Genomics (ACMG) genetic variant classification criteria and guidelines. The detected *GSDME* mutation was verified by Sanger sequencing. Sequence alignment was performed after quality control of sequencing data to analyze whether the detected variants were consistent with co-segregation of hearing phenotypes and genotypes within the family. As for controls, DNA samples from 100 unrelated normal hearing individuals were also collected.

### Reverse transcription polymerase chain reaction (RT-PCR) analysis

To observe the potential effect of this mutation on mRNA level,10ml of fresh peripheral venous blood was drawn from each family member, total RNA was extracted from peripheral blood, and RT-PCR was performed to synthesize the cDNA of *GSDME* by reverse transcription. A forward primer from exon 7 (5’–AACAGACAGCTTTGAGTGACA–3’) and a reverse primer from exon 10(5’–ATCCCAAACCTTTCTGTATCT–3’) was designed, followed by PCR amplification of cDNA fragments in this region. After the above PCR products were purified, we performed sequencing analysis; and the sequencing results were compared with the standard sequences(https://blast.ncbi.nlm.nih.gov/Blast.cgi).

### Bioinformatics analysis

In order to investigate the potential mechanism of deafness caused by the *GSDME* gene mutation and explore its involvement in the molecular pathways, we downloaded the gene expression profile microarray data GSE70167 from the GEO database of NCBI (https://www.ncbi.nlm.nih.gov/geo/query/acc.cgi?acc=GSE70167). The dataset was uploaded by Ken Op de Beeck in 2015 and included two HEK293T transfected cell populations: wild type and *GSDME*-knockout (KO) type. The knockout of the *GSDME* gene may affect the expression of specific associated genes and may impact molecular pathways. So we performed Gene Set Enrichment Analysis (GSEA) (v.4.2.3) to explore the signaling pathways between the two groups. The expression level of the *GSDME* gene was used as the phenotype label. The reference gene set was the c2.cp.kegg.v7.5.1.symbols.gmt, and the NES (normalized enrichment score) was calculated. Gene set permutations were performed 1,000 times for each analysis to identify significantly different pathways. The gene set was significantly enriched if the normal p-value <0.05 and FDR (false discovery rate) q value <25%.

## Results

### Clinical findings

The family exhibited a typical autosomal dominant inheritance pattern of hearing loss, which consists of 5 generations, 37 people (Fig 1). These affected members did not differ by gender and revealed post-lingual, bilateral, symmetrical, progressive, and sensorineural hearing loss. The onset of hearing impairment ranges from 25 to 35 years. Most patients had discomfort with intermittent tinnitus, none had vertigo or balance disorders, and their intelligence and communication were normal. None of the patients had a clear history of aminoglycoside application or noise exposure before the onset of the disease. No abnormalities of other organ systems were seen on physical examination. A high-resolution CT scan of the temporal bone showed no abnormal findings. The genealogical analysis showed that the deafness phenotype was transmitted for four consecutive generations, consistent with autosomal dominant inheritance, except for the younger 5th generation.

**Fig 2:**
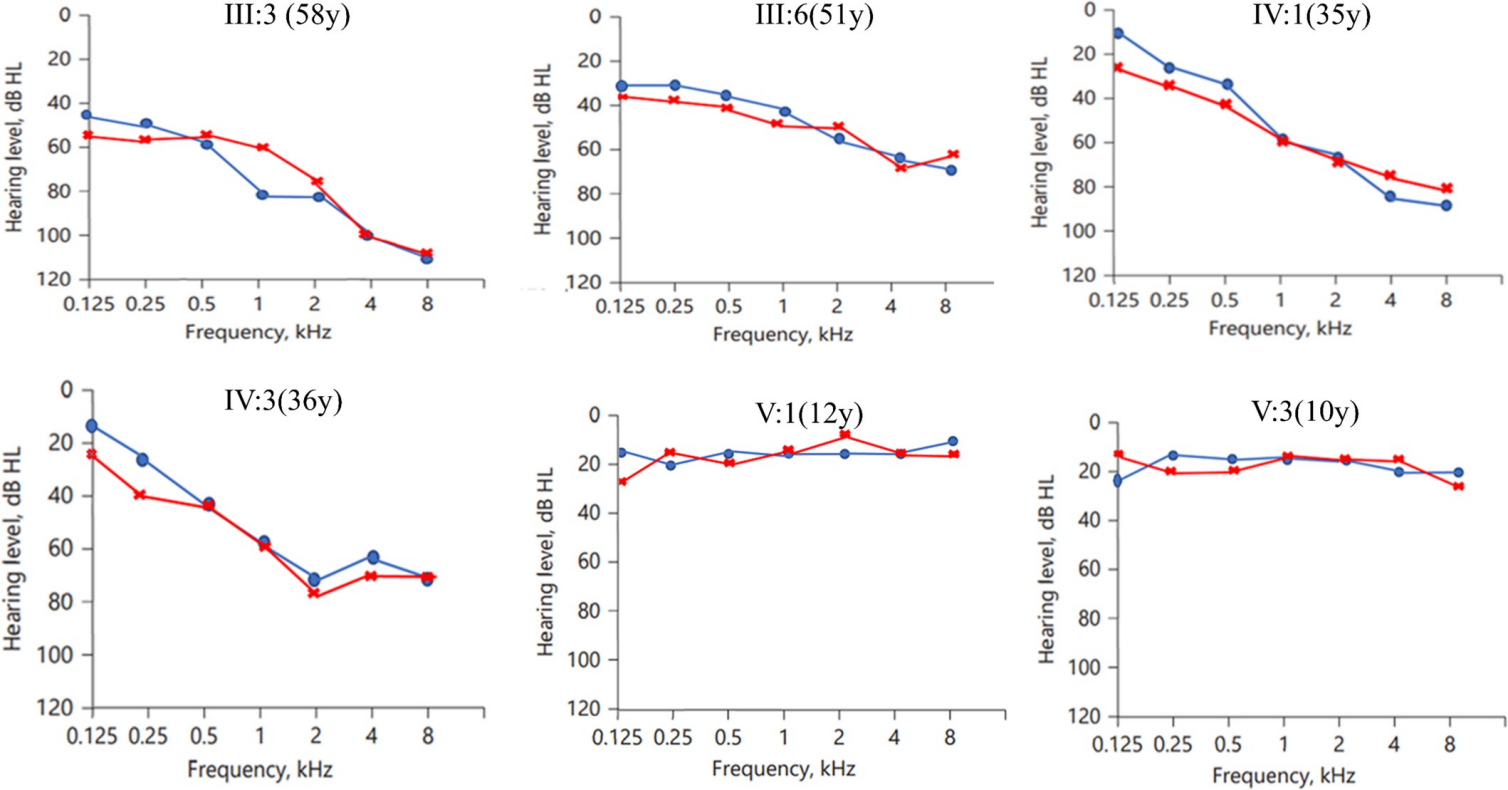
Pure-tone audiometry for affected individuals. Audiograms were obtained with air conduction with the frequency range of 0.125 kHz to 8 kHz. Symbols “o” and “x” denotes air conduction pure-tone thresholds at different right and left ear frequencies.

Pure-tone audiograms of five patients showed moderate to severe sensorineural hearing loss bilaterally (Table 1). The hearing curves of the affected individuals showed that the low-frequency hearing was less impaired and got progressively worse with age, while the high-frequency hearing was more impaired at the beginning (Fig 2). The hearing loss began in the high frequencies and gradually progressed to all frequencies. Although a *GSDME* mutation was confirmed at a subsequent mutation screen, a young member of 10 years old (V:3) still exhibited a regular hearing curve pattern because the age of onset was not reached.

**Table 1:** Summary of clinical data for the family members.

Subjects	Gender	Age (years)		PTA (dB HL)*		Degree of hearing loss	Audiogram shape	Genotype
		At testing	At onset	Left	Right			
III-2	Female	61	28	73.50	76.10	Severe	Down-slopping	DelTTC/WT
III-3	Male	58	25	72.50	83.75	Severe	Down-slopping	DelTTC/WT
III-4	Female	56	-	23.45	22.15	Normal	Flat	WT/WT
III-6	Male	51	35	47.50	55.00	Moderate	Down-slopping	DelTTC/WT
IV-1	Male	35	25	47.50	45.00	Moderate	Down-slopping	DelTTC/WT
IV-2	Female	33	-	18.05	12.55	Normal	Flat	WT/WT
IV-3	Male	36	26	55.00	50.00	Moderate	Down-slopping	DelTTC/WT
V-1	Female	12	-	15.00	16.25	Normal	Flat	WT/WT
V-2	Male	13	-	16.25	17.50	Normal	Flat	WT/WT
V-3	Male	10	-	17.50	15.00	Normal	Flat	DelTTC/WT

*The PTA was calculated from audiometric thresholds at 0.5, 1, 2, and 4 kHz. The severity of HL was categorized as follows: Mild (PTA ≤40 dB), moderate (40 dB < PTA ≤70 dB), severe (70 dB < PTA ≤90 dB), and profound (PTA >90 dB). PTA: Pure-tone average.

**Fig 3:**
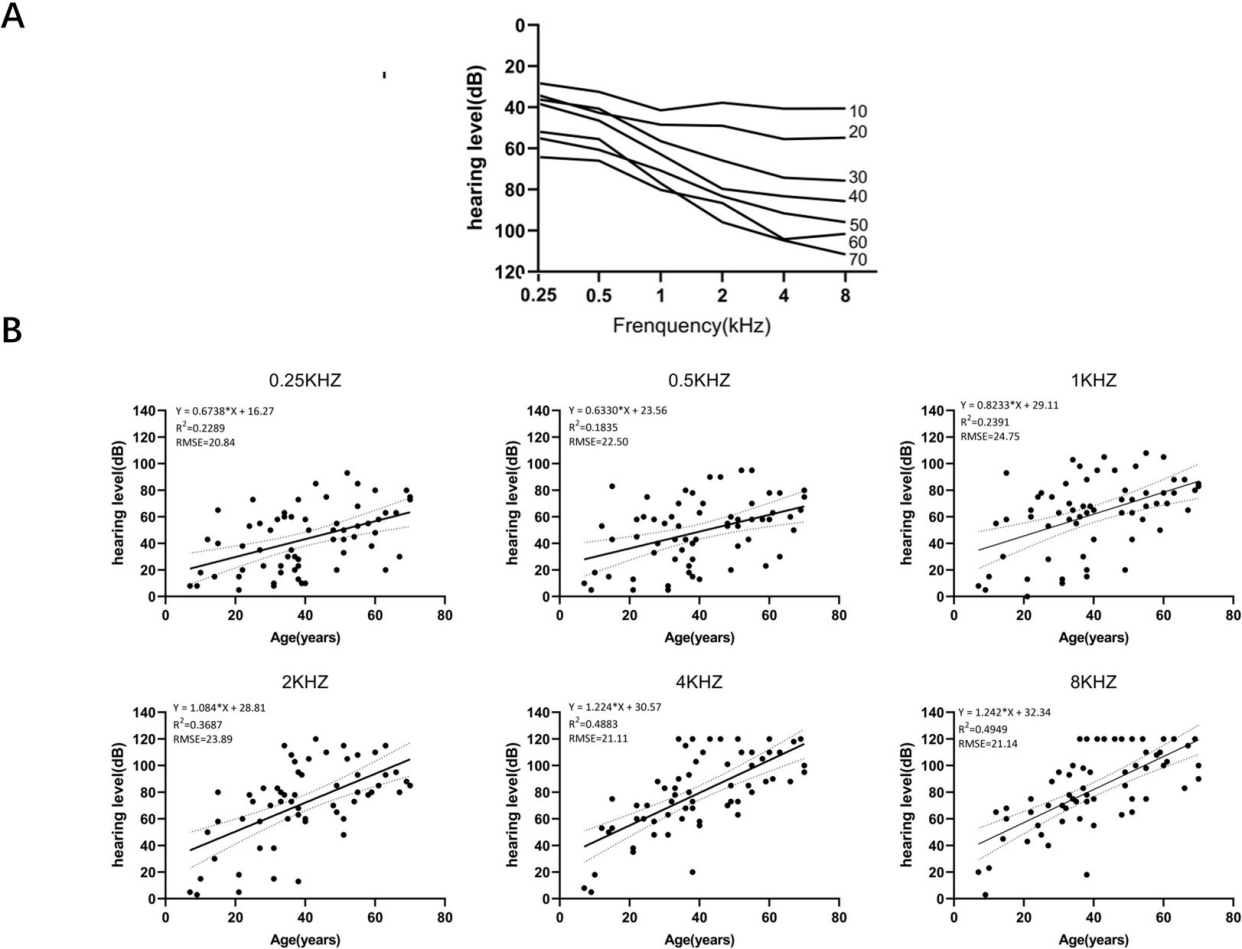
Audiological characteristics of *GSDME*-related hearing loss. (A) ARTA for patients with pathogenic variants. (B) ATD: Rate of hearing loss deterioration in PTA.

Including the results of this study, we divided the collected data into three groups: this study, delTTC variant, and other variants. We found there was no significant difference in the progression rates for each frequency (0.25 kHz, 0.5 kHz, 1 kHz, 2 kHz, 4 kHz, and 8 kHz) between the three groups (P>0.05; Table 2). Therefore, we combined the three datasets and calculated the pooled ATD, which ranged from 0.63 to 1.24 dB per year (Fig 3B). We found that the difference between each frequency was statistically significant (p<0.05), with the ATD growing as the frequency increased. ARTA (Fig 3A) confirms the down-sloping audiometric configuration, which starts as mild at low and mid frequencies and moderates at high frequencies. It progresses with age and becomes moderate to severe at mid frequencies and profound at high frequencies.

**Table 2:** Progression rates by frequency between the three groups.

	0.25kHz	0.5kHz	1kHz	2kHz	4kHz	8kHz
This study	0.7312	0.7917	0.9210	1.116	1.450	1.514
DelTTC	0.8667	0.8139	1.339	1.697	1.550	1.522
Others	0.5711	0.5028	0.5791	0.8050	1.026	1.026
p-value	0.7300	0.7017	0.2232	0.1029	0.3193	0.2852

### Identification of *GSDME* mutation (c.991-15_991_13delTTC)

**Fig 4:**
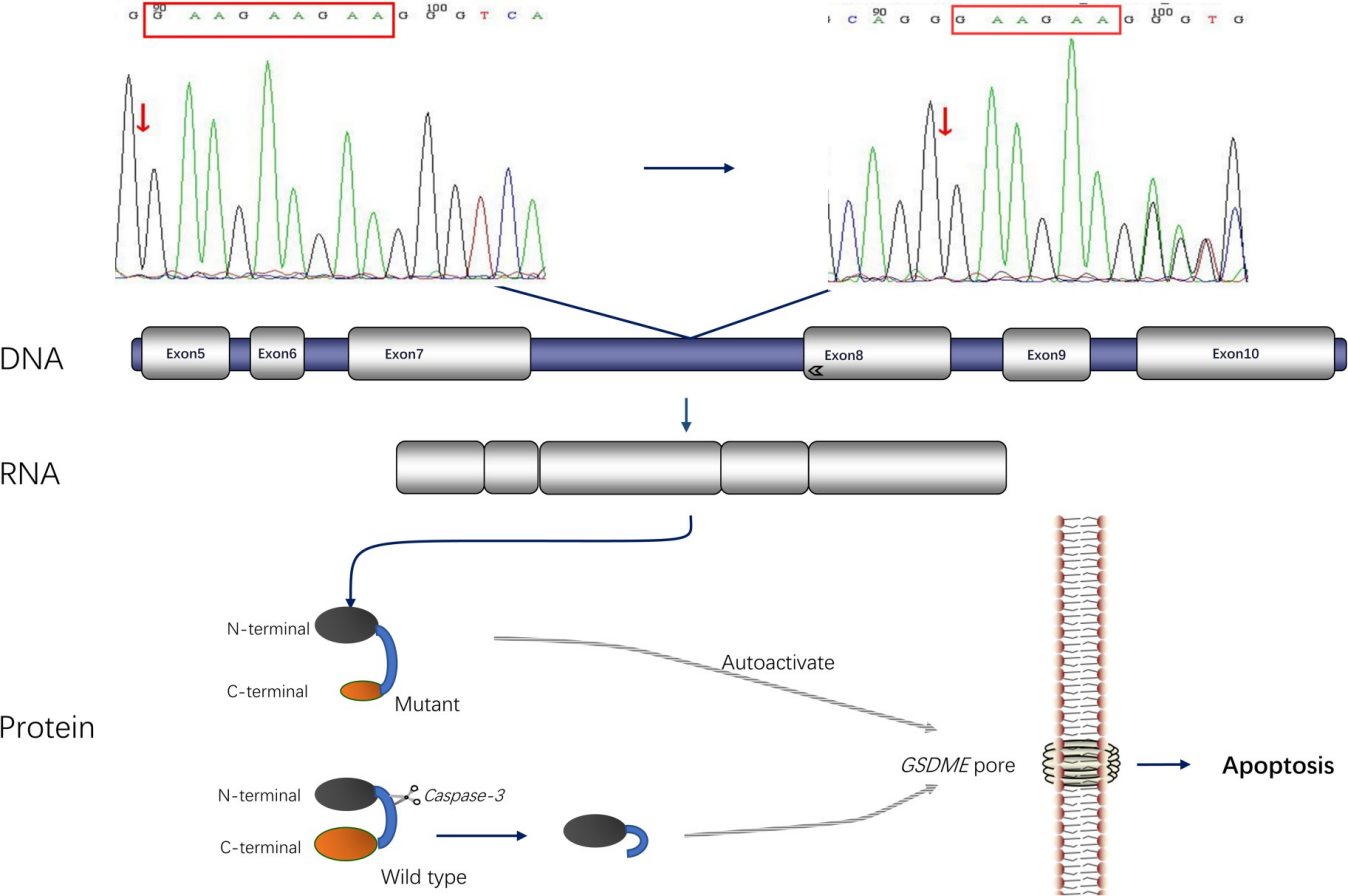
Chromatogram of the genomic structure of *GSDME* and the location of this mutation (c. 991–15_991-13del). Skipping of exon 8 can truncate the C-terminal domain of mutant *GSDME*, uncovering the apoptosis-inducing region, leading to apoptosis. On the other hand, caspase-3 can cleave wild-type *GSDME* to generate an active N-terminal that also forms pores in the plasma membrane and permeabilizes it.

We excluded mutations in the mitochondrial DNA and miRNA regions and compared the measured nuclear gene sequences with the human genome reference sequence (GRCh37/hg19). The average sequencing depth on the targeted region was 505.10-fold, with 97.25% of the region covered by at least 20×, demonstrating the high quality of the sequencing data. Our study covered 751 loci for 147 genes in the deep intron region reported by HGMD. It increased probe coverage density for 29 genes (full-length design for GJB2, SLC26A4, and POU3F4 genes) to facilitate Cap-CNV analysis and detection of deafness-associated genes in mitochondrial loop DNA. The results were all negative. After variant filtering and further prioritization for genes associated with ADNSHL, the mutation in the *GSDME* gene was found in the proband. A 3-bp deletion (c.991-15_991-13delTTC, NM_004403) was identified in intron 7 (Fig 4). This mutation was verified by Sanger sequencing in the other patients. The mutation is co-segregated with hearing loss in this family. However, the mutation was absent in 100 age-matched unrelated random controls with normal hearings. According to the American College of Medical Genetics and Genomics (ACMG) 2015 guidelines, the variant is classified as pathogenic, with the applied criteria of PS3_M, PS4_M, PM2, PP1_S, and PP3.

### Validation of skipping of exon 8 in *GSDME*

We designed the primers from exon 7 and exon 10 to amplify the cDNA fragment. Sequencing analysis of the patient’s RT-PCR product revealed a deletion of the exon 8 sequence (exon skipping) in his mutant transcript, resulting in a direct linkage between exon 7 and exon 9 sequences (Fig 4). No mutations in any part of the mRNA were detected by RT-PCR with other primers.

### Bioinformatics findings

**Fig 5:**
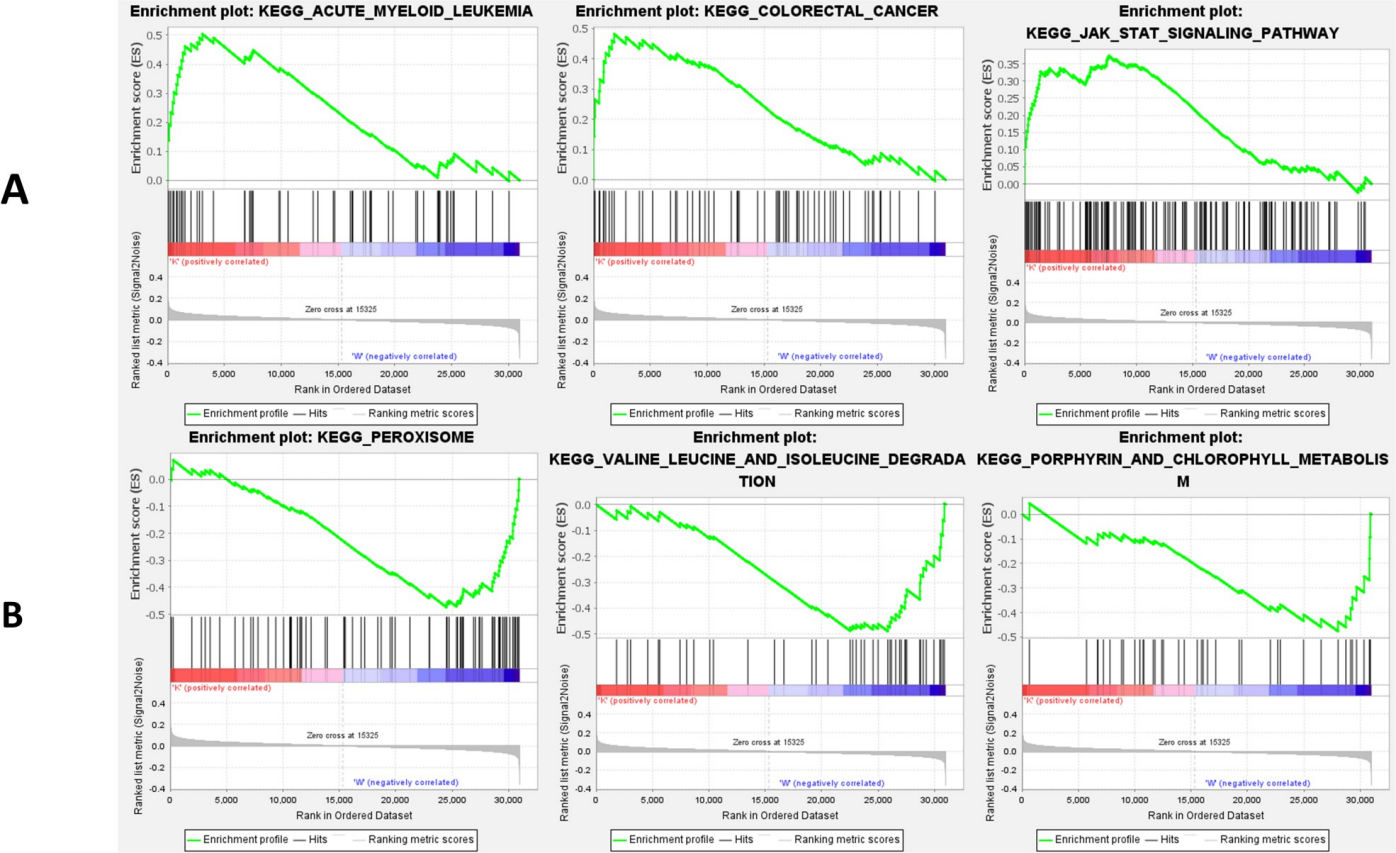
Significant KEGG enrichment plot from GSEA analysis. A: The top three upregulated pathways; B: The top three downregulated pathways.

In view of NES, FDR q-value, and nominal p-value, significantly enriched signaling pathways were selected (S3 File). Compared with the WT group, our GSEA results (Fig 5) indicated that *GSDME* knockout upregulated acute myeloid leukemia, colorectal cancer, JAK/STAT signaling pathway, and hedgehog signaling pathway. We also found a significant downregulation in 11 signaling pathways involved in the peroxisome, valine leucine and isoleucine degradation, porphyrin and chlorophyll metabolism, pyruvate metabolism, selenoamino acid metabolism, base excision repair, propanoate metabolism, glycine serine and threonine metabolism, DNA replication, lysine degradation, and pyrimidine metabolism.

## Discussion

Hereditary hearing loss has been described as one of the diseases with a high degree of genetic heterogeneity, which is reflected in the diversity and complexity of deafness phenotypes, inheritance patterns, and pathogenic genes. In the present study, the graphical pedigree analysis exhibited that the inheritance pattern of deafness in this family is non-syndromic autosomal dominant, with late-onset, post-lingual, and progressive hearing loss. We found that the patients have an early onset of high-frequency hearing loss, and the decline was particularly significant between 1 and 8 kHz. Meanwhile, we observed that a young patient (V:3) shows no symptoms on all frequencies, which have no impact on hearing or speaking. This demonstrates that the clinical phenotype of this family is generally consistent with that of other *GSDME* deafness families reported in the literature [17].

We searched PubMed for all reported *GSDME* mutations that can lead to non-syndromic hearing loss. We found at least 23 *GSDME* families worldwide, including 6 from Europe and 14 from Asia (Table 3). Fourteen mutation types were identified, most located in introns 7 or 8. Significantly, all known pathogenic variants of *GSDME* resulted in the skipping of exon 8. In this study, a *GSDME* mutation (c.991-15_991-13delTTC) was detected in the family and reported in several East Asian families [9–11,18,19]. These findings suggest that *GSDME* mutations may be ethnically specific and that the c.991-15_991-13del mutation may be a more recurrent causative factor in autosomal dominant hearing loss in East Asians.

**Table 3:** Summary of all reported *GSDME* variants leading to hearing loss.

Variant	Effect of variant	Location	Chromosomal location	Ethnicity	Onset age (year old)	Hearing impairment	References
c.990 +503_ 990 +1691 del ins132	Skipping of exon 8	Intron 7	chr7:24746055- 24747243delins	Dutch	5–15	High frequency	Van Laer(1998) [4]
c.991 − 15_991- 13delTTC	Skipping of exon 8	Intron 7	chr7:24746007–24746010	Chinese	7–30	High frequency	Yu(2003) [18]
				Korean	20	High frequency	Park(2010) [19]
				Japanese	10–30	High frequency	Nishio (2014) [14]
				Japanese	18	High frequency	Nishio (2014) [14]
				Chinese	6–20	All frequency	Wang(2018) [10]
				East Asian	8–18	Moderate-high frequency	Booth(2018) [11]
				European	10–15	High frequency	Booth(2020) [9]
				Chinese	25–35	High frequency	This study
c.991-6 C>G	Skipping of exon 8	Intron 7	chr7:24746001	Dutch	28–49	High-all frequency	Bischoff (2004) [17]
IVS8+4 A>G	Skipping of exon 8	Intron 8	chr7:24745799	Chinese	11–50	High-all frequency	Cheng(2007) [20]
c.991-2 A>G	Skipping of exon 8	Intron 7	chr7:24745997	Chinese	8–18	High frequency	Chai(2014) [21]
				European	10	High-all frequency	Booth(2018) [11]
IVS8+1 delG	Skipping of exon 8	Intron 8	chr7:24745801–24745802	Chinese	8–30	High-all frequency	Li(2015) [13]
				Chinese	12–30	High-all frequency	Wang(2021) [22]
c.1183 G > C	Skipping of exon 8	Exon 8	chr7:24745803	Chinese			Chen(2016) [23]
c.666_669delCTAC				Italian		Moderate-high frequency	Morgan(2018) [24]
c.1102 C>G	Skipping of exon 8	Exon 8	chr7:24745884	European		High frequency	Booth(2018) [11]
c.1154 C> T	Skipping of exon 8	Exon 8	chr7:24745832	Iranian		High frequency	Booth(2018) [11]
c.991-3 C> A	Skipping of exon 8	Intron 7	chr7:24745998	Chinese	20–39	High frequency	Wang(2018) [10]
c.991-1 G>C	Skipping of exon 8	Intron 7	chr7:24745996	Chinese	10–40	High-all frequency	Chen(2020) [8]
c.1183+1 G>C	Skipping of exon 8	Intron 8	chr7:24745802	Chinese	18–25	High-all frequency	Li(2022) [25]
IVS7-2 A > G	Skipping of exon 8	Intron 7	chr7: 24698355–24795539	Chinese	13–61	High-all frequency	Jin(2022) [26]

We also collected and analyzed pure-tone hearing threshold profiles of deafness families with *GSDME* mutation based on all available data previously reported, given the mechanism associated with all *GSDME*-related hearing loss. Progression at every frequency was fairly constant in the first two decades, in contrast to a more rapid progression in the third decade and particularly pronounced at high frequencies (Fig 3A). A study of inner ear histopathology [12] found that patients with sensorineural hearing loss caused by the *GSDME* variant suffered from varying degrees of loss of inner and outer cochlear hair cells. Different areas of the cochlear basilar membrane sense different frequencies of sound, with the base of the cochlea for high frequencies and the apex of the cochlea for low frequencies [27]. In addition, the hair cells of the basilar membrane at the base of the cochlea have lower levels of antioxidant enzymes than the apex of the cochlea, making them more susceptible to ototoxic substances [28]. Hearing impairment is a highly heterogeneous disorder with environmental and genetic causes [29]. Splicing efficiency, ethnic background, and modifier genes [30] may be responsible for phenotypic variation. However, we compared ATDs between the three groups and found no significant differences in hearing thresholds at each frequency. Further studies involving larger samples are warranted to confirm the correlation between these data.

The *GSDME* gene contains 10 exons and encodes a protein consisting of 496 amino acids. Skipping of exon 8 led to a frame-shift in coding sequences, resulting in changes of 41 amino acids (residuals 331–371) and in-frame deletion of 126 amino acids in the reading frame. The final effect produces a truncated protein with a stop codon at position 372 [4]. The bioinformatics predictions are consistent with the experimental results [31] that the N terminal is an important region for the function of this family of proteins. The structural variation of the C-terminal affects the complementarity of the C- and N-terminal domains. The mutant GSDME protein has a novel function, probably due to the truncated C-terminal and exposure of the N-terminal domain (Fig 4).

Recent studies have shown that GSDME is mainly expressed in the placenta, brain, heart, kidney, and other human tissues, such as cochlear hair cells [32]. The cochlear hair cells may be more susceptible to the toxicity of the truncated proteins or have insufficient degradability, and a significant accumulation of the abnormal protein causes non-syndromic deafness [33]. Rogers et al found that the GSDME protein can regulate the necrosis and catabolism of apoptotic cells [31]. It has been hypothesized that mutant GSDME is associated with a specific membrane protein that accumulates on the cell plasma membrane to produce a triggering effect that causes apoptosis (Fig 4), which may be an essential part of the pathogenesis of GSDME. Most scholars now favor the specific "gain-of-function mutation" hypothesis to explain the molecular mechanism of deafness caused by *GSDME* mutation [34]. The mutant GSDME protein generated by the skipping of exon 8 does not cause deafness due to haploinsufficiency, but rather the mutant protein is endowed with a new function that has toxic effects on the auditory system. Gregan transfected yeast cells with the human mutant GSDME protein, resulting in a significant decrease in the activity of the transfected cells. In contrast, yeast cells transfected with the wild-type GSDME protein had normal viability [35]. In *GSDME* knockout mice, only an increase in the number of the fourth row of cochlear hair cells was observed. No frequency-specific ABR examinations or vestibular function abnormalities were observed [36]. The results of these experiments also support the above hypothesis.

In combination with its association with deafness, it was found that the expression level of GSDME in tumor cells was significantly lower than that in normal cells, and enhanced GSDME expression had an inhibitory effect on the proliferation of hepatocellular carcinoma cells. The intrinsic mechanism was related to the apoptotic ability of GSDME [37]. Therefore, the GSDME mutant protein may modify the apoptotic process in hearing-associated cells, which leads to hearing loss [38]. It has been shown that caspase-3 cleaves GSDME to release the N-terminal during apoptosis [31], which can cause secondary necrosis or pyroptosis. Upregulation of different cytochrome c oxidase (COX) genes is associated with cell death mechanisms under oxidative stress in a mutant GSDME cell line [39]. Downregulation of protein sorting and folding-related mechanisms in the same study model suggested a potential role of endoplasmic reticulum stress in the cell death induced by *GSDME*. These indicated that all these contributing factors, individually or interactively, led to apoptosis and cochlear damage.

Knockout of *the GSDME* gene may upregulate or downregulate some signaling pathways. *GSDME* is the first gene known to be involved in monogenic apoptotic deafness, and it is also a tumor suppressor gene with a critical role in major types of tumors. Although its knockout affects the signaling pathways in acute myeloid leukemia and colorectal cancer, many studies are still needed to confirm the association of GSDME-related hearing loss with the development of cancer and protection from cancer. The JAK-STAT signaling pathway takes part in many biological processes, including cell division, apoptosis, and immune regulation [40]. It plays an essential role during the embryonic development of the inner ear, and it is involved in progenitor cell proliferation and differentiation as well as the cell fate decision [41]. Peroxisome function together with mitochondria in several essential biochemical pathways, such as autophagy [42]^.^ A study also found [43] peroxisome could provide an antioxidant defense triggered by noise exposure in hair cells and auditory neurons of the inner ear. *GSDME* was related to “valine leucine and isoleucine degradation” as our GSEA results. These three amino acids belong to the proteinogenic branched-chain amino acids (BCAAs), which have a possible pathophysiological relationship with human iron metabolism and are mediated by the mechanistic target of the rapamycin complex 1 (mTORC1) pathway [44]. The chlorophyll metabolism pathway acts as a key player in erythropoiesis by regulating erythroid heme synthesis [45]. All of these provide indications for further studies on the pathogenesis of deafness with the *GSDME* variant.

In conclusion, we described a five-generation Chinese family with late-onset, progressive, non-syndromic, sensorineural hearing loss. In this family, a *GSDME* mutation (c.991-15_991-13delTTC) leading to the skipping of exon 8 was identified through targeted genomic sequencing and co-segregation analysis, which was considered to be a hotspot mutation in East Asian populations. The reported *GSDME* mutations responsible for progressive hearing loss affect high and mid frequencies earlier and more severely than low frequencies. Further mechanistic studies are needed to better understand the role mutant *GSDME* plays in hearing loss.

## Supporting information

S1 Table The 415 genes screened in this study. (DOCX)

S1 Fig Sequencing results. (TIF)

S1 File Available data was collected in families with reported non-syndromic deafness with *GSDME* mutation. (XLSX)

S2 File The original data of this study. (XLSX)

S3 File GSEA analysis. (ZIP)
