# Supplementary material for: Mutation analysis of the GSDME gene in a Chinese family with non-syndromic hearing loss
Source: PLoS One. 2022 Nov 9;17(11):e0276233. doi: 10.1371/journal.pone.0276233 (PMC9645625; doi:10.1371/journal.pone.0276233)
Supplement: S1 Table — (DOCX) [file pone.0276233.s001.docx]

| *ABCD1* | *ABHD12* | *ABHD5* | *ACO2* | *ACOX1* | *ACTB* | *ACTG1* | *ADCY1* |
| --- | --- | --- | --- | --- | --- | --- | --- |
| *ADGRV1* | *AIFM1* | *AK2* | *ALMS1* | *ALX3* | *ALX4* | *AMER1* | *ANKH* |
| *ANKRD11* | *AP1S1* | *ARSE* | *ASPA* | *ATP1A3* | *ATP2B2* | *ATP6V1B1* | *ATP6V1B2* |
| *ATRX* | *BCAP31* | *BCOR* | *BCS1L* | *BDP1* | *BEAN1* | *BMP1* | *BRAF* |
| *BSND* | *BTD* | *CABP2* | *CACNA1D* | *CATSPER2* | *CCDC50* | *CD151* | *CD164* |
| *CDC14A* | *CDH23* | *CEACAM1*  *6* | *CHD7* | *CHM* | *CHSY1* | *CIB2* | *CIDEA* |
| *CISD2* | *CLCN7* | *CLCNKA* | *CLCNKB* | *CLDN14* | *CLIC5* | *CLPP* | *CLRN1* |
| *COCH* | *COL11A1* | *COL11A2* | *COL1A1* | *COL1A2* | *COL2A1* | *COL4A3* | *COL4A4* |
| *COL4A5* | *COL4A6* | *COL9A1* | *COL9A2* | *COLEC10* | *COLEC11* | *COQ6* | *COX6B1* |
| *CRTAP* | *CRYM* | *DCDC2* | *DCHS1* | *DDX11* | *DHODH* | *DIABLO* | *DIAPH1* |
| *DIAPH3* | *DLX5* | *DMP1* | *DMXL2* | *DNA2* | *DNAJC17* | *DNAJC3* | *DNMT1* |
| *DSPP* | *DVL1* | *ECM1* | *EDN1* | *EDN3* | *EDNRA* | *EDNRB* | *EFTUD2* |
| *EHMT1* | *ELAC2* | *ELMOD3* | *ENPP1* | *EPS8* | *EPS8L2* | *ERAL1* | *ERCC6* |
| *ERCC8* | *ESPN* | *ESRP1* | *ESRRB* | *EYA1* | *EYA4* | *FAT4* | *FGF10* |
| *FGF3* | *FGF8* | *FGF9* | *FGFR1* | *FGFR2* | *FGFR3* | *FKBP10* | *FKBP14* |
| *FLNA* | *FLNB* | *FLVCR2* | *FOXC1* | *FOXI1* | *FREM1* | *FUCA1* | *FXN* |
| *GAB1* | *GALE* | *GATA3* | *GDF3* | *GDF5* | *GDF6* | *GFER* | *GIPC3* |
| *GJA1* | *GJB1* | *GJB2* | *GJB3* | *GJB6* | *GLYAT* | *GMPPA* | *GMPPB* |
| *GNAI3* | *GPC3* | *GPSM2* | *GRHL2* | *GRXCR1* | *GRXCR2* | *GSC* | *GSDME* |
| *GSTP1* | *GSTT1* | *GUCY2D* | *HARS* | *HARS2* | *HGF* | *HMX1* | *HOMER2* |
| *HOXA1* | *HOXA11* | *HOXA2* | *HOXB1* | *HPD* | *HSD17B10* | *HSD17B4* | *HSPA1A* |
| *HSPA1L* | *HSPA2* | *IARS2* | *IDS* | *IFITM5* | *IFNLR1* | *IGF1* | *IL13* |
| *ILDR1* | *IRX5* | *ITM2B* | *KARS* | *KAT6B* | *KCNE1* | *KCNJ10* | *KCNQ1* |
| *KCNQ4* | *KITLG* | *KRT9* | *LAMA3* | *LARS2* | *LHFPL5* | *LHX3* | *LMNA* |
| *LMX1A* | *LOXHD1* | *LRP2* | *LRP4* | *LRTOMT* | *MAF* | *MAN2B1* | *MANBA* |
| *MARVELD*  *2* | *MASP1* | *MCM2* | *MEOX1* | *MET* | *MFN2* | *MGP* | *MIR182* |
| *MIR183* | *MIR96* | *MITF* | *MPZ* | *MPZL2* | *MSRB3* | *MT-RNR1* | *MT-TA* |
| *MT-TC* | *MT-TE* | *MT-TF* | *MT-TH* | *MT-TI* | *MT-TK* | *MT-TL1* | *MT-TP* |
| *MT-TQ* | *MT-TR* | *MT-TS1* | *MT-TS2* | *MT-TT* | *MT-TW* | *MYH14* | *MYH9* |
| *MYO15A* | *MYO1A* | *MYO1E* | *MYO3A* | *MYO6* | *MYO7A* | *NARS2* | *NDP* |
| *NDRG1* | *NEFL* | *NELL2* | *NF2* | *NLRP3* | *NOG* | *NOP56* | *NSD1* |
| *OFD1* | *OPA1* | *OSBPL2* | *OSTM1* | *OTOA* | *OTOF* | *OTOG* | *OTOGL* |
| *P2RX2* | *P3H1* | *PABPN1* | *PAX1* | *PAX2* | *PAX3* | *PCDH15* | *PCDH9* |
| *PCNA* | *PDE1C* | *PDSS1* | *PDSS2* | *PDZD7* | *PEX1* | *PEX10* | *PEX11B* |
| *PEX12* | *PEX13* | *PEX14* | *PEX16* | *PEX19* | *PEX2* | *PEX26* | *PEX3* |
| *PEX5* | *PEX6* | *PEX7* | *PHYH* | *PIGL* | *PJVK* | *PLCB4* | *PLEKHM1* |
| *PLOD3* | *PLP1* | *PMP22* | *PNPLA8* | *PNPT1* | *POLD1* | *POLG* | *POLG2* |

**Supplementary Table 1: The 415 genes screened in this study**

| *POLR1C* | *POLR1D* | *POU3F4* | *POU4F3* | *PPIB* | *PPIP5K2* | *PQBP1* | *PROK2* |
| --- | --- | --- | --- | --- | --- | --- | --- |
| *PROKR2* | *PRPS1* | *PRRX1* | *PTPN11* | *PTPRQ* | *PTPRR* | *RAB23* | *RAB40AL* |
| *RAF1* | *RDX* | *RECQL4* | *REST* | *RIPOR2* | *RNASEH1* | *RNASET2* | *ROR1* |
| *RPGR* | *RPS6KA3* | *RRM2B* | *S1PR2* | *SALL1* | *SALL4* | *SCO1* | *SEC23A* |
| *SEMA3E* | *SERAC1* | *SERPINB6* | *SERPINF1* | *SERPINH1* | *SETBP1* | *SIX1* | *SIX5* |
| *SLC17A8* | *SLC19A2* | *SLC22A4* | *SLC25A4* | *SLC26A4* | *SLC26A5* | *SLC29A3* | *SLC33A1* |
| *SLC44A4* | *SLC4A11* | *SLC52A2* | *SLC52A3* | *SLC9A1* | *SLITRK6* | *SMAD4* | *SMARCA4* |
| *SMARCB1* | *SMPX* | *SNAI2* | *SNX10* | *SOD1* | *SOD2* | *SOST* | *SOX10* |
| *SOX2* | *SOX9* | *SPARC* | *SPTBN4* | *SQSTM1* | *ST3GAL5* | *STRC* | *SUCLA2* |
| *SUCLG1* | *SYNE4* | *TBC1D24* | *TBX22* | *TCIRG1* | *TCOF1* | *TECTA* | *TFAP2A* |
| *THOC1* | *THRB* | *TIMM8A* | *TJP2* | *TMC1* | *TMEM126A* | *TMEM132E* | *TMIE* |
| *TMPRSS3* | *TMPRSS4* | *TNC* | *TNFRSF11*  *A* | *TNFRSF11*  *B* | *TNFSF11* | *TP63* | *TPRN* |
| *TRIOBP* | *TRMU* | *TSHZ1* | *TSPEAR* | *TUBB4A* | *TUBB4B* | *TWIST1* | *TWNK* |
| *TXNL4A* | *TYMP* | *TYR* | *UBR1* | *UGT1A1* | *UQCC2* | *USH1C* | *USH1G* |
| *USH2A* | *VHL* | *WBP2* | *WFS1* | *WHRN* | *XPNPEP3* | *YAP1* |  |
