# Supplementary figures and images for: Mutation analysis of the GSDME gene in a Chinese family with non-syndromic hearing loss
Source: PLoS One. 2022 Nov 9;17(11):e0276233. doi: 10.1371/journal.pone.0276233 (PMC9645625; doi:10.1371/journal.pone.0276233)

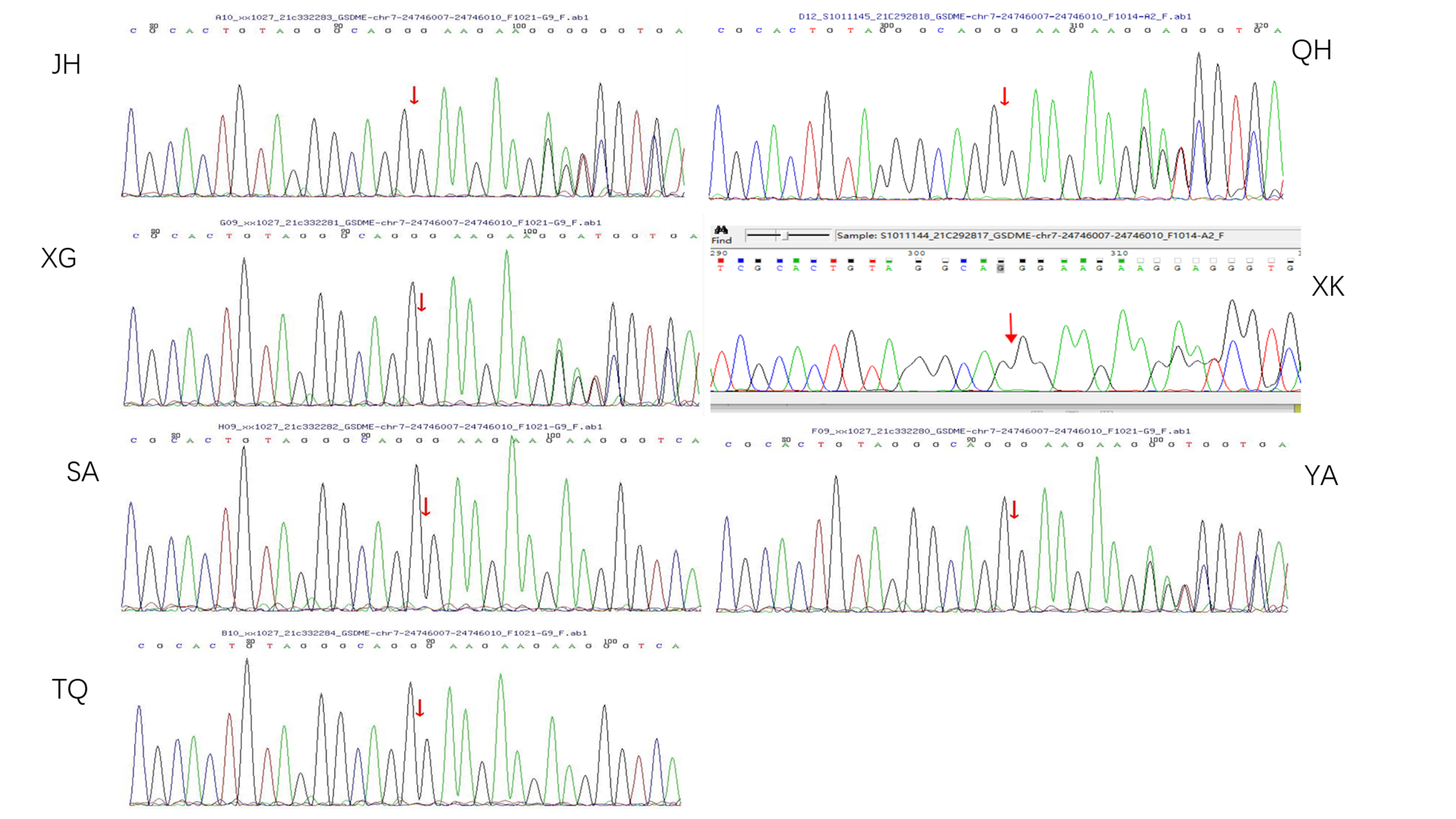

Supplement: S1 Fig — (TIF) [file pone.0276233.s002.tif]
