# Supplementary material for: Mutation analysis of the GSDME gene in a Chinese family with non-syndromic hearing loss
Source: PLoS One. 2022 Nov 9;17(11):e0276233. doi: 10.1371/journal.pone.0276233 (PMC9645625; doi:10.1371/journal.pone.0276233)
Supplement: S3 File — (ZIP) [file pone.0276233.s005.zip › S3_File/Gsea/gsea_report_for_K_1659660506975.html]

Report for K 1659660506975 [GSEA]

| GS  follow link to MSigDB | GS DETAILS | SIZE | ES | NES | NOM p-val | FDR q-val | FWER p-val | RANK AT MAX | LEADING EDGE || 1 | KEGG\_ACUTE\_MYELOID\_LEUKEMIA | Details ... | 56 | 0.50 | 1.96 | 0.000 | 0.049 | 0.038 | 3070 | tags=27%, list=10%, signal=30% |
| 2 | KEGG\_COLORECTAL\_CANCER | Details ... | 62 | 0.48 | 1.83 | 0.000 | 0.112 | 0.164 | 1825 | tags=18%, list=6%, signal=19% |
| 3 | KEGG\_JAK\_STAT\_SIGNALING\_PATHWAY | Details ... | 149 | 0.37 | 1.70 | 0.002 | 0.239 | 0.437 | 7530 | tags=34%, list=24%, signal=45% |
| 4 | KEGG\_STARCH\_AND\_SUCROSE\_METABOLISM | Details ... | 50 | 0.44 | 1.66 | 0.006 | 0.253 | 0.555 | 7986 | tags=50%, list=26%, signal=67% |
| 5 | KEGG\_HEDGEHOG\_SIGNALING\_PATHWAY | Details ... | 56 | 0.43 | 1.64 | 0.010 | 0.230 | 0.608 | 6113 | tags=30%, list=20%, signal=38% |
| 6 | KEGG\_NOTCH\_SIGNALING\_PATHWAY | Details ... | 46 | 0.43 | 1.57 | 0.014 | 0.327 | 0.798 | 6648 | tags=41%, list=21%, signal=53% |
| 7 | KEGG\_LEISHMANIA\_INFECTION | Details ... | 68 | 0.39 | 1.55 | 0.006 | 0.317 | 0.843 | 10096 | tags=51%, list=33%, signal=76% |
| 8 | KEGG\_PATHWAYS\_IN\_CANCER | Details ... | 318 | 0.31 | 1.54 | 0.002 | 0.303 | 0.867 | 4846 | tags=22%, list=16%, signal=25% |
| 9 | KEGG\_WNT\_SIGNALING\_PATHWAY | Details ... | 148 | 0.34 | 1.53 | 0.006 | 0.297 | 0.898 | 6207 | tags=28%, list=20%, signal=34% |
| 10 | KEGG\_REGULATION\_OF\_ACTIN\_CYTOSKELETON | Details ... | 202 | 0.32 | 1.53 | 0.000 | 0.270 | 0.899 | 7293 | tags=32%, list=24%, signal=41% |
| 11 | KEGG\_T\_CELL\_RECEPTOR\_SIGNALING\_PATHWAY | Details ... | 103 | 0.35 | 1.51 | 0.014 | 0.270 | 0.919 | 7412 | tags=29%, list=24%, signal=38% |
| 12 | KEGG\_P53\_SIGNALING\_PATHWAY | Details ... | 64 | 0.38 | 1.49 | 0.024 | 0.283 | 0.946 | 1825 | tags=17%, list=6%, signal=18% |
| 13 | KEGG\_GLYCOSPHINGOLIPID\_BIOSYNTHESIS\_GANGLIO\_SERIES | Details ... | 15 | 0.53 | 1.48 | 0.053 | 0.287 | 0.955 | 1297 | tags=20%, list=4%, signal=21% |
| 14 | KEGG\_O\_GLYCAN\_BIOSYNTHESIS | Details ... | 25 | 0.46 | 1.46 | 0.063 | 0.299 | 0.960 | 6473 | tags=44%, list=21%, signal=56% |
| 15 | KEGG\_MAPK\_SIGNALING\_PATHWAY | Details ... | 252 | 0.30 | 1.45 | 0.009 | 0.304 | 0.968 | 3738 | tags=19%, list=12%, signal=22% |
| 16 | KEGG\_PROGESTERONE\_MEDIATED\_OOCYTE\_MATURATION | Details ... | 81 | 0.35 | 1.43 | 0.046 | 0.321 | 0.979 | 5297 | tags=23%, list=17%, signal=28% |
| 17 | KEGG\_GAP\_JUNCTION | Details ... | 82 | 0.35 | 1.43 | 0.041 | 0.318 | 0.981 | 6957 | tags=35%, list=22%, signal=45% |
| 18 | KEGG\_VASCULAR\_SMOOTH\_MUSCLE\_CONTRACTION | Details ... | 109 | 0.33 | 1.41 | 0.036 | 0.334 | 0.987 | 9046 | tags=40%, list=29%, signal=57% |
| 19 | KEGG\_CHEMOKINE\_SIGNALING\_PATHWAY | Details ... | 181 | 0.30 | 1.39 | 0.013 | 0.364 | 0.996 | 7349 | tags=29%, list=24%, signal=38% |
| 20 | KEGG\_ALLOGRAFT\_REJECTION | Details ... | 36 | 0.40 | 1.38 | 0.082 | 0.360 | 0.996 | 9881 | tags=58%, list=32%, signal=86% |
| 21 | KEGG\_ADIPOCYTOKINE\_SIGNALING\_PATHWAY |  | 64 | 0.35 | 1.37 | 0.055 | 0.367 | 0.999 | 2231 | tags=22%, list=7%, signal=24% |
| 22 | KEGG\_PRION\_DISEASES |  | 35 | 0.40 | 1.36 | 0.092 | 0.364 | 0.999 | 4230 | tags=20%, list=14%, signal=23% |
| 23 | KEGG\_VEGF\_SIGNALING\_PATHWAY |  | 73 | 0.34 | 1.35 | 0.087 | 0.377 | 1.000 | 7405 | tags=34%, list=24%, signal=45% |
| 24 | KEGG\_CELL\_CYCLE |  | 110 | 0.32 | 1.35 | 0.038 | 0.367 | 1.000 | 2218 | tags=14%, list=7%, signal=15% |
| 25 | KEGG\_ADHERENS\_JUNCTION |  | 67 | 0.33 | 1.33 | 0.083 | 0.386 | 1.000 | 6209 | tags=33%, list=20%, signal=41% |
| 26 | KEGG\_ASTHMA |  | 29 | 0.41 | 1.32 | 0.121 | 0.405 | 1.000 | 9881 | tags=59%, list=32%, signal=86% |
| 27 | KEGG\_TIGHT\_JUNCTION |  | 122 | 0.30 | 1.31 | 0.053 | 0.396 | 1.000 | 8859 | tags=38%, list=29%, signal=53% |
| 28 | KEGG\_FRUCTOSE\_AND\_MANNOSE\_METABOLISM |  | 32 | 0.39 | 1.30 | 0.137 | 0.408 | 1.000 | 1655 | tags=19%, list=5%, signal=20% |
| 29 | KEGG\_CHRONIC\_MYELOID\_LEUKEMIA |  | 72 | 0.33 | 1.30 | 0.076 | 0.394 | 1.000 | 2231 | tags=17%, list=7%, signal=18% |
| 30 | KEGG\_LEUKOCYTE\_TRANSENDOTHELIAL\_MIGRATION |  | 108 | 0.31 | 1.29 | 0.065 | 0.403 | 1.000 | 5440 | tags=28%, list=18%, signal=34% |
| 31 | KEGG\_GLYCOSPHINGOLIPID\_BIOSYNTHESIS\_LACTO\_AND\_NEOLACTO\_SERIES |  | 25 | 0.41 | 1.29 | 0.143 | 0.402 | 1.000 | 5506 | tags=32%, list=18%, signal=39% |
| 32 | KEGG\_SMALL\_CELL\_LUNG\_CANCER |  | 83 | 0.31 | 1.29 | 0.083 | 0.390 | 1.000 | 2179 | tags=18%, list=7%, signal=19% |
| 33 | KEGG\_RNA\_DEGRADATION |  | 54 | 0.35 | 1.28 | 0.117 | 0.397 | 1.000 | 7105 | tags=30%, list=23%, signal=38% |
| 34 | KEGG\_MELANOGENESIS |  | 101 | 0.30 | 1.28 | 0.089 | 0.385 | 1.000 | 5922 | tags=26%, list=19%, signal=32% |
| 35 | KEGG\_COMPLEMENT\_AND\_COAGULATION\_CASCADES |  | 68 | 0.32 | 1.27 | 0.113 | 0.398 | 1.000 | 5486 | tags=29%, list=18%, signal=36% |
| 36 | KEGG\_BASAL\_CELL\_CARCINOMA |  | 55 | 0.33 | 1.26 | 0.122 | 0.410 | 1.000 | 6101 | tags=27%, list=20%, signal=34% |
| 37 | KEGG\_FOCAL\_ADHESION |  | 192 | 0.26 | 1.25 | 0.060 | 0.426 | 1.000 | 5965 | tags=24%, list=19%, signal=29% |
| 38 | KEGG\_GLYCOSAMINOGLYCAN\_BIOSYNTHESIS\_CHONDROITIN\_SULFATE |  | 21 | 0.41 | 1.22 | 0.209 | 0.466 | 1.000 | 3362 | tags=24%, list=11%, signal=27% |
| 39 | KEGG\_CYTOKINE\_CYTOKINE\_RECEPTOR\_INTERACTION |  | 253 | 0.25 | 1.22 | 0.065 | 0.465 | 1.000 | 7494 | tags=28%, list=24%, signal=37% |
| 40 | KEGG\_SYSTEMIC\_LUPUS\_ERYTHEMATOSUS |  | 55 | 0.32 | 1.22 | 0.154 | 0.456 | 1.000 | 10076 | tags=51%, list=33%, signal=75% |
| 41 | KEGG\_ENDOCYTOSIS |  | 172 | 0.26 | 1.21 | 0.110 | 0.467 | 1.000 | 6316 | tags=27%, list=20%, signal=34% |
| 42 | KEGG\_STEROID\_BIOSYNTHESIS |  | 15 | 0.44 | 1.20 | 0.208 | 0.473 | 1.000 | 2810 | tags=27%, list=9%, signal=29% |
| 43 | KEGG\_HEMATOPOIETIC\_CELL\_LINEAGE |  | 86 | 0.29 | 1.20 | 0.166 | 0.475 | 1.000 | 7614 | tags=37%, list=25%, signal=49% |
| 44 | KEGG\_VIRAL\_MYOCARDITIS |  | 67 | 0.30 | 1.19 | 0.175 | 0.474 | 1.000 | 9613 | tags=45%, list=31%, signal=65% |
| 45 | KEGG\_TASTE\_TRANSDUCTION |  | 48 | 0.33 | 1.19 | 0.176 | 0.464 | 1.000 | 6233 | tags=27%, list=20%, signal=34% |
| 46 | KEGG\_ALDOSTERONE\_REGULATED\_SODIUM\_REABSORPTION |  | 42 | 0.34 | 1.19 | 0.207 | 0.461 | 1.000 | 4717 | tags=26%, list=15%, signal=31% |
| 47 | KEGG\_PENTOSE\_PHOSPHATE\_PATHWAY |  | 26 | 0.37 | 1.17 | 0.236 | 0.492 | 1.000 | 2375 | tags=15%, list=8%, signal=17% |
| 48 | KEGG\_GNRH\_SIGNALING\_PATHWAY |  | 98 | 0.28 | 1.17 | 0.183 | 0.496 | 1.000 | 9263 | tags=43%, list=30%, signal=61% |
| 49 | KEGG\_B\_CELL\_RECEPTOR\_SIGNALING\_PATHWAY |  | 73 | 0.30 | 1.16 | 0.167 | 0.495 | 1.000 | 1115 | tags=8%, list=4%, signal=9% |
| 50 | KEGG\_PROXIMAL\_TUBULE\_BICARBONATE\_RECLAMATION |  | 23 | 0.37 | 1.16 | 0.240 | 0.491 | 1.000 | 3374 | tags=30%, list=11%, signal=34% |
| 51 | KEGG\_TYPE\_II\_DIABETES\_MELLITUS |  | 43 | 0.33 | 1.16 | 0.211 | 0.495 | 1.000 | 4820 | tags=28%, list=16%, signal=33% |
| 52 | KEGG\_VASOPRESSIN\_REGULATED\_WATER\_REABSORPTION |  | 44 | 0.31 | 1.15 | 0.244 | 0.497 | 1.000 | 5297 | tags=23%, list=17%, signal=27% |
| 53 | KEGG\_OOCYTE\_MEIOSIS |  | 104 | 0.27 | 1.14 | 0.216 | 0.525 | 1.000 | 7020 | tags=32%, list=23%, signal=41% |
| 54 | KEGG\_AXON\_GUIDANCE |  | 127 | 0.26 | 1.14 | 0.202 | 0.518 | 1.000 | 1642 | tags=9%, list=5%, signal=10% |
| 55 | KEGG\_CELL\_ADHESION\_MOLECULES\_CAMS |  | 127 | 0.25 | 1.12 | 0.233 | 0.548 | 1.000 | 5440 | tags=23%, list=18%, signal=28% |
| 56 | KEGG\_AUTOIMMUNE\_THYROID\_DISEASE |  | 51 | 0.30 | 1.11 | 0.286 | 0.557 | 1.000 | 9881 | tags=51%, list=32%, signal=75% |
| 57 | KEGG\_NITROGEN\_METABOLISM |  | 23 | 0.36 | 1.11 | 0.318 | 0.549 | 1.000 | 320 | tags=13%, list=1%, signal=13% |
| 58 | KEGG\_NEUROACTIVE\_LIGAND\_RECEPTOR\_INTERACTION |  | 260 | 0.23 | 1.11 | 0.201 | 0.552 | 1.000 | 7072 | tags=28%, list=23%, signal=36% |
| 59 | KEGG\_STEROID\_HORMONE\_BIOSYNTHESIS |  | 54 | 0.30 | 1.11 | 0.280 | 0.543 | 1.000 | 8835 | tags=35%, list=29%, signal=49% |
| 60 | KEGG\_AMYOTROPHIC\_LATERAL\_SCLEROSIS\_ALS |  | 51 | 0.30 | 1.10 | 0.301 | 0.541 | 1.000 | 1825 | tags=12%, list=6%, signal=12% |
| 61 | KEGG\_CALCIUM\_SIGNALING\_PATHWAY |  | 167 | 0.24 | 1.10 | 0.235 | 0.538 | 1.000 | 7126 | tags=31%, list=23%, signal=40% |
| 62 | KEGG\_FC\_GAMMA\_R\_MEDIATED\_PHAGOCYTOSIS |  | 93 | 0.27 | 1.10 | 0.274 | 0.530 | 1.000 | 6293 | tags=28%, list=20%, signal=35% |
| 63 | KEGG\_GRAFT\_VERSUS\_HOST\_DISEASE |  | 39 | 0.31 | 1.10 | 0.302 | 0.535 | 1.000 | 9881 | tags=54%, list=32%, signal=79% |
| 64 | KEGG\_TGF\_BETA\_SIGNALING\_PATHWAY |  | 84 | 0.27 | 1.09 | 0.308 | 0.533 | 1.000 | 4670 | tags=24%, list=15%, signal=28% |
| 65 | KEGG\_PHENYLALANINE\_METABOLISM |  | 17 | 0.39 | 1.09 | 0.331 | 0.543 | 1.000 | 7910 | tags=47%, list=26%, signal=63% |
| 66 | KEGG\_PANCREATIC\_CANCER |  | 69 | 0.28 | 1.08 | 0.316 | 0.541 | 1.000 | 2845 | tags=17%, list=9%, signal=19% |
| 67 | KEGG\_MELANOMA |  | 71 | 0.27 | 1.08 | 0.311 | 0.537 | 1.000 | 7349 | tags=32%, list=24%, signal=42% |
| 68 | KEGG\_NATURAL\_KILLER\_CELL\_MEDIATED\_CYTOTOXICITY |  | 133 | 0.24 | 1.08 | 0.288 | 0.531 | 1.000 | 7494 | tags=32%, list=24%, signal=41% |
| 69 | KEGG\_LONG\_TERM\_POTENTIATION |  | 69 | 0.27 | 1.08 | 0.313 | 0.533 | 1.000 | 9263 | tags=43%, list=30%, signal=62% |
| 70 | KEGG\_THYROID\_CANCER |  | 29 | 0.33 | 1.08 | 0.337 | 0.527 | 1.000 | 1437 | tags=14%, list=5%, signal=14% |
| 71 | KEGG\_PROSTATE\_CANCER |  | 88 | 0.26 | 1.07 | 0.320 | 0.529 | 1.000 | 7349 | tags=27%, list=24%, signal=36% |
| 72 | KEGG\_DORSO\_VENTRAL\_AXIS\_FORMATION |  | 23 | 0.34 | 1.06 | 0.385 | 0.551 | 1.000 | 6776 | tags=39%, list=22%, signal=50% |
| 73 | KEGG\_LONG\_TERM\_DEPRESSION |  | 66 | 0.27 | 1.04 | 0.370 | 0.584 | 1.000 | 9046 | tags=38%, list=29%, signal=53% |
| 74 | KEGG\_RIBOSOME |  | 87 | 0.25 | 1.04 | 0.376 | 0.578 | 1.000 | 11488 | tags=40%, list=37%, signal=64% |
| 75 | KEGG\_RENAL\_CELL\_CARCINOMA |  | 66 | 0.27 | 1.04 | 0.411 | 0.588 | 1.000 | 7349 | tags=27%, list=24%, signal=36% |
| 76 | KEGG\_ARACHIDONIC\_ACID\_METABOLISM |  | 56 | 0.27 | 1.03 | 0.403 | 0.594 | 1.000 | 2685 | tags=16%, list=9%, signal=18% |
| 77 | KEGG\_GLYCOSAMINOGLYCAN\_BIOSYNTHESIS\_HEPARAN\_SULFATE |  | 24 | 0.33 | 1.02 | 0.420 | 0.601 | 1.000 | 9889 | tags=63%, list=32%, signal=92% |
| 78 | KEGG\_NEUROTROPHIN\_SIGNALING\_PATHWAY |  | 123 | 0.23 | 1.01 | 0.452 | 0.630 | 1.000 | 7377 | tags=28%, list=24%, signal=37% |
| 79 | KEGG\_ALZHEIMERS\_DISEASE |  | 138 | 0.23 | 1.01 | 0.432 | 0.626 | 1.000 | 2310 | tags=12%, list=7%, signal=12% |
| 80 | KEGG\_EPITHELIAL\_CELL\_SIGNALING\_IN\_HELICOBACTER\_PYLORI\_INFECTION |  | 64 | 0.26 | 1.00 | 0.454 | 0.626 | 1.000 | 9905 | tags=44%, list=32%, signal=64% |
| 81 | KEGG\_N\_GLYCAN\_BIOSYNTHESIS |  | 45 | 0.28 | 1.00 | 0.450 | 0.623 | 1.000 | 5607 | tags=24%, list=18%, signal=30% |
| 82 | KEGG\_TYPE\_I\_DIABETES\_MELLITUS |  | 42 | 0.28 | 0.99 | 0.439 | 0.641 | 1.000 | 9881 | tags=50%, list=32%, signal=73% |
| 83 | KEGG\_SPHINGOLIPID\_METABOLISM |  | 35 | 0.29 | 0.98 | 0.467 | 0.663 | 1.000 | 5950 | tags=31%, list=19%, signal=39% |
| 84 | KEGG\_GALACTOSE\_METABOLISM |  | 25 | 0.31 | 0.98 | 0.481 | 0.657 | 1.000 | 2033 | tags=20%, list=7%, signal=21% |
| 85 | KEGG\_INTESTINAL\_IMMUNE\_NETWORK\_FOR\_IGA\_PRODUCTION |  | 46 | 0.27 | 0.97 | 0.500 | 0.666 | 1.000 | 9881 | tags=46%, list=32%, signal=67% |
| 86 | KEGG\_TYROSINE\_METABOLISM |  | 39 | 0.27 | 0.97 | 0.482 | 0.664 | 1.000 | 7910 | tags=36%, list=26%, signal=48% |
| 87 | KEGG\_ENDOMETRIAL\_CANCER |  | 52 | 0.26 | 0.96 | 0.491 | 0.675 | 1.000 | 4330 | tags=17%, list=14%, signal=20% |
| 88 | KEGG\_INSULIN\_SIGNALING\_PATHWAY |  | 134 | 0.21 | 0.95 | 0.556 | 0.687 | 1.000 | 7377 | tags=29%, list=24%, signal=38% |
| 89 | KEGG\_OLFACTORY\_TRANSDUCTION |  | 375 | 0.19 | 0.94 | 0.649 | 0.715 | 1.000 | 6060 | tags=21%, list=20%, signal=26% |
| 90 | KEGG\_BASAL\_TRANSCRIPTION\_FACTORS |  | 34 | 0.27 | 0.92 | 0.579 | 0.758 | 1.000 | 5243 | tags=24%, list=17%, signal=28% |
| 91 | KEGG\_GLYCEROPHOSPHOLIPID\_METABOLISM |  | 69 | 0.23 | 0.92 | 0.596 | 0.752 | 1.000 | 3066 | tags=16%, list=10%, signal=18% |
| 92 | KEGG\_MATURITY\_ONSET\_DIABETES\_OF\_THE\_YOUNG |  | 21 | 0.30 | 0.92 | 0.546 | 0.748 | 1.000 | 6298 | tags=33%, list=20%, signal=42% |
| 93 | KEGG\_ERBB\_SIGNALING\_PATHWAY |  | 85 | 0.22 | 0.91 | 0.609 | 0.756 | 1.000 | 3097 | tags=13%, list=10%, signal=14% |
| 94 | KEGG\_UBIQUITIN\_MEDIATED\_PROTEOLYSIS |  | 128 | 0.21 | 0.90 | 0.684 | 0.772 | 1.000 | 7812 | tags=32%, list=25%, signal=43% |
| 95 | KEGG\_APOPTOSIS |  | 86 | 0.22 | 0.90 | 0.632 | 0.766 | 1.000 | 8107 | tags=29%, list=26%, signal=39% |
| 96 | KEGG\_GLYCEROLIPID\_METABOLISM |  | 42 | 0.25 | 0.90 | 0.632 | 0.766 | 1.000 | 3712 | tags=19%, list=12%, signal=22% |
| 97 | KEGG\_PURINE\_METABOLISM |  | 143 | 0.20 | 0.89 | 0.695 | 0.769 | 1.000 | 5715 | tags=21%, list=18%, signal=26% |
| 98 | KEGG\_AMINOACYL\_TRNA\_BIOSYNTHESIS |  | 21 | 0.29 | 0.88 | 0.586 | 0.781 | 1.000 | 10313 | tags=48%, list=33%, signal=71% |
| 99 | KEGG\_ALPHA\_LINOLENIC\_ACID\_METABOLISM |  | 18 | 0.30 | 0.87 | 0.648 | 0.807 | 1.000 | 8915 | tags=44%, list=29%, signal=62% |
| 100 | KEGG\_ALANINE\_ASPARTATE\_AND\_GLUTAMATE\_METABOLISM |  | 30 | 0.27 | 0.87 | 0.634 | 0.800 | 1.000 | 5737 | tags=27%, list=19%, signal=33% |
| 101 | KEGG\_INOSITOL\_PHOSPHATE\_METABOLISM |  | 51 | 0.23 | 0.83 | 0.764 | 0.875 | 1.000 | 5256 | tags=22%, list=17%, signal=26% |
| 102 | KEGG\_METABOLISM\_OF\_XENOBIOTICS\_BY\_CYTOCHROME\_P450 |  | 69 | 0.21 | 0.82 | 0.780 | 0.877 | 1.000 | 3037 | tags=9%, list=10%, signal=10% |
| 103 | KEGG\_LINOLEIC\_ACID\_METABOLISM |  | 28 | 0.25 | 0.82 | 0.725 | 0.879 | 1.000 | 9383 | tags=46%, list=30%, signal=67% |
| 104 | KEGG\_FC\_EPSILON\_RI\_SIGNALING\_PATHWAY |  | 77 | 0.20 | 0.81 | 0.821 | 0.880 | 1.000 | 7412 | tags=27%, list=24%, signal=36% |
| 105 | KEGG\_ARRHYTHMOGENIC\_RIGHT\_VENTRICULAR\_CARDIOMYOPATHY\_ARVC |  | 72 | 0.20 | 0.80 | 0.829 | 0.888 | 1.000 | 5089 | tags=19%, list=16%, signal=23% |
| 106 | KEGG\_VIBRIO\_CHOLERAE\_INFECTION |  | 53 | 0.21 | 0.79 | 0.811 | 0.894 | 1.000 | 9642 | tags=42%, list=31%, signal=60% |
| 107 | KEGG\_RETINOL\_METABOLISM |  | 62 | 0.20 | 0.79 | 0.849 | 0.889 | 1.000 | 9383 | tags=34%, list=30%, signal=48% |
| 108 | KEGG\_HUNTINGTONS\_DISEASE |  | 149 | 0.17 | 0.79 | 0.947 | 0.883 | 1.000 | 6378 | tags=19%, list=21%, signal=24% |
| 109 | KEGG\_DILATED\_CARDIOMYOPATHY |  | 88 | 0.19 | 0.78 | 0.916 | 0.888 | 1.000 | 5297 | tags=18%, list=17%, signal=22% |
| 110 | KEGG\_NOD\_LIKE\_RECEPTOR\_SIGNALING\_PATHWAY |  | 54 | 0.20 | 0.75 | 0.894 | 0.923 | 1.000 | 7048 | tags=26%, list=23%, signal=33% |
| 111 | KEGG\_ASCORBATE\_AND\_ALDARATE\_METABOLISM |  | 25 | 0.24 | 0.74 | 0.818 | 0.919 | 1.000 | 7799 | tags=32%, list=25%, signal=43% |
| 112 | KEGG\_MISMATCH\_REPAIR |  | 23 | 0.23 | 0.70 | 0.871 | 0.951 | 1.000 | 1627 | tags=13%, list=5%, signal=14% |
| 113 | KEGG\_PHOSPHATIDYLINOSITOL\_SIGNALING\_SYSTEM |  | 73 | 0.18 | 0.70 | 0.948 | 0.944 | 1.000 | 6035 | tags=23%, list=19%, signal=29% |
| 114 | KEGG\_PATHOGENIC\_ESCHERICHIA\_COLI\_INFECTION |  | 51 | 0.18 | 0.68 | 0.954 | 0.948 | 1.000 | 6403 | tags=25%, list=21%, signal=32% |
Table: Gene sets enriched in phenotype **K (6 samples)**[plain text format]****

  
