# Supplementary material for: Mutation analysis of the GSDME gene in a Chinese family with non-syndromic hearing loss
Source: PLoS One. 2022 Nov 9;17(11):e0276233. doi: 10.1371/journal.pone.0276233 (PMC9645625; doi:10.1371/journal.pone.0276233)
Supplement: S3 File — (ZIP) [file pone.0276233.s005.zip › S3_File/Gsea/gsea_report_for_W_1659660506975.html]

Report for W 1659660506975 [GSEA]

| GS  follow link to MSigDB | GS DETAILS | SIZE | ES | NES | NOM p-val | FDR q-val | FWER p-val | RANK AT MAX | LEADING EDGE || 1 | KEGG\_PEROXISOME | Details ... | 75 | -0.47 | -1.92 | 0.000 | 0.021 | 0.028 | 6495 | tags=45%, list=21%, signal=57% |
| 2 | KEGG\_VALINE\_LEUCINE\_AND\_ISOLEUCINE\_DEGRADATION | Details ... | 43 | -0.49 | -1.76 | 0.000 | 0.074 | 0.183 | 5130 | tags=44%, list=17%, signal=53% |
| 3 | KEGG\_PORPHYRIN\_AND\_CHLOROPHYLL\_METABOLISM | Details ... | 39 | -0.48 | -1.68 | 0.006 | 0.114 | 0.380 | 2964 | tags=23%, list=10%, signal=25% |
| 4 | KEGG\_PYRUVATE\_METABOLISM | Details ... | 39 | -0.47 | -1.66 | 0.009 | 0.103 | 0.432 | 7501 | tags=46%, list=24%, signal=61% |
| 5 | KEGG\_SELENOAMINO\_ACID\_METABOLISM | Details ... | 24 | -0.51 | -1.64 | 0.017 | 0.102 | 0.508 | 3180 | tags=21%, list=10%, signal=23% |
| 6 | KEGG\_BASE\_EXCISION\_REPAIR | Details ... | 32 | -0.46 | -1.62 | 0.017 | 0.100 | 0.566 | 7129 | tags=47%, list=23%, signal=61% |
| 7 | KEGG\_PROPANOATE\_METABOLISM | Details ... | 31 | -0.46 | -1.58 | 0.020 | 0.113 | 0.668 | 8249 | tags=52%, list=27%, signal=70% |
| 8 | KEGG\_GLYCINE\_SERINE\_AND\_THREONINE\_METABOLISM | Details ... | 31 | -0.46 | -1.58 | 0.016 | 0.106 | 0.685 | 3796 | tags=29%, list=12%, signal=33% |
| 9 | KEGG\_DNA\_REPLICATION | Details ... | 35 | -0.46 | -1.57 | 0.014 | 0.099 | 0.702 | 8563 | tags=51%, list=28%, signal=71% |
| 10 | KEGG\_PRIMARY\_BILE\_ACID\_BIOSYNTHESIS | Details ... | 16 | -0.53 | -1.52 | 0.051 | 0.128 | 0.826 | 7134 | tags=50%, list=23%, signal=65% |
| 11 | KEGG\_LYSINE\_DEGRADATION | Details ... | 39 | -0.43 | -1.52 | 0.019 | 0.117 | 0.827 | 6441 | tags=44%, list=21%, signal=55% |
| 12 | KEGG\_BIOSYNTHESIS\_OF\_UNSATURATED\_FATTY\_ACIDS | Details ... | 20 | -0.49 | -1.50 | 0.068 | 0.126 | 0.878 | 3907 | tags=35%, list=13%, signal=40% |
| 13 | KEGG\_PYRIMIDINE\_METABOLISM | Details ... | 90 | -0.35 | -1.48 | 0.016 | 0.129 | 0.898 | 4275 | tags=23%, list=14%, signal=27% |
| 14 | KEGG\_GLYCOSYLPHOSPHATIDYLINOSITOL\_GPI\_ANCHOR\_BIOSYNTHESIS | Details ... | 25 | -0.46 | -1.47 | 0.068 | 0.131 | 0.920 | 10680 | tags=64%, list=34%, signal=98% |
| 15 | KEGG\_AMINO\_SUGAR\_AND\_NUCLEOTIDE\_SUGAR\_METABOLISM | Details ... | 41 | -0.38 | -1.37 | 0.074 | 0.243 | 0.997 | 4032 | tags=29%, list=13%, signal=34% |
| 16 | KEGG\_PROTEIN\_EXPORT | Details ... | 22 | -0.44 | -1.33 | 0.112 | 0.300 | 1.000 | 7852 | tags=36%, list=25%, signal=49% |
| 17 | KEGG\_CYSTEINE\_AND\_METHIONINE\_METABOLISM | Details ... | 34 | -0.38 | -1.31 | 0.119 | 0.324 | 1.000 | 2497 | tags=15%, list=8%, signal=16% |
| 18 | KEGG\_CITRATE\_CYCLE\_TCA\_CYCLE | Details ... | 30 | -0.37 | -1.28 | 0.136 | 0.373 | 1.000 | 7365 | tags=27%, list=24%, signal=35% |
| 19 | KEGG\_PANTOTHENATE\_AND\_COA\_BIOSYNTHESIS | Details ... | 16 | -0.43 | -1.26 | 0.178 | 0.400 | 1.000 | 7667 | tags=50%, list=25%, signal=66% |
| 20 | KEGG\_HYPERTROPHIC\_CARDIOMYOPATHY\_HCM | Details ... | 81 | -0.30 | -1.23 | 0.142 | 0.432 | 1.000 | 9445 | tags=44%, list=30%, signal=64% |
| 21 | KEGG\_BETA\_ALANINE\_METABOLISM |  | 22 | -0.39 | -1.20 | 0.209 | 0.490 | 1.000 | 8249 | tags=50%, list=27%, signal=68% |
| 22 | KEGG\_FATTY\_ACID\_METABOLISM |  | 39 | -0.34 | -1.19 | 0.206 | 0.509 | 1.000 | 8486 | tags=49%, list=27%, signal=67% |
| 23 | KEGG\_PENTOSE\_AND\_GLUCURONATE\_INTERCONVERSIONS |  | 27 | -0.36 | -1.18 | 0.213 | 0.505 | 1.000 | 1923 | tags=19%, list=6%, signal=20% |
| 24 | KEGG\_DRUG\_METABOLISM\_OTHER\_ENZYMES |  | 49 | -0.31 | -1.16 | 0.232 | 0.542 | 1.000 | 2601 | tags=14%, list=8%, signal=16% |
| 25 | KEGG\_PRIMARY\_IMMUNODEFICIENCY |  | 35 | -0.32 | -1.14 | 0.283 | 0.569 | 1.000 | 7857 | tags=43%, list=25%, signal=57% |
| 26 | KEGG\_OXIDATIVE\_PHOSPHORYLATION |  | 96 | -0.27 | -1.12 | 0.229 | 0.600 | 1.000 | 9201 | tags=38%, list=30%, signal=53% |
| 27 | KEGG\_ANTIGEN\_PROCESSING\_AND\_PRESENTATION |  | 85 | -0.27 | -1.12 | 0.235 | 0.584 | 1.000 | 3379 | tags=15%, list=11%, signal=17% |
| 28 | KEGG\_HOMOLOGOUS\_RECOMBINATION |  | 24 | -0.36 | -1.11 | 0.301 | 0.587 | 1.000 | 7638 | tags=42%, list=25%, signal=55% |
| 29 | KEGG\_OTHER\_GLYCAN\_DEGRADATION |  | 15 | -0.38 | -1.08 | 0.339 | 0.661 | 1.000 | 1011 | tags=20%, list=3%, signal=21% |
| 30 | KEGG\_LYSOSOME |  | 118 | -0.24 | -1.05 | 0.340 | 0.725 | 1.000 | 3712 | tags=16%, list=12%, signal=18% |
| 31 | KEGG\_NON\_SMALL\_CELL\_LUNG\_CANCER |  | 54 | -0.27 | -1.04 | 0.384 | 0.760 | 1.000 | 4123 | tags=22%, list=13%, signal=26% |
| 32 | KEGG\_MTOR\_SIGNALING\_PATHWAY |  | 50 | -0.27 | -1.03 | 0.376 | 0.743 | 1.000 | 4374 | tags=22%, list=14%, signal=26% |
| 33 | KEGG\_BUTANOATE\_METABOLISM |  | 34 | -0.30 | -1.03 | 0.398 | 0.724 | 1.000 | 8599 | tags=47%, list=28%, signal=65% |
| 34 | KEGG\_NICOTINATE\_AND\_NICOTINAMIDE\_METABOLISM |  | 20 | -0.34 | -1.03 | 0.405 | 0.706 | 1.000 | 4858 | tags=35%, list=16%, signal=41% |
| 35 | KEGG\_CYTOSOLIC\_DNA\_SENSING\_PATHWAY |  | 53 | -0.26 | -1.03 | 0.397 | 0.689 | 1.000 | 3279 | tags=17%, list=11%, signal=19% |
| 36 | KEGG\_RIG\_I\_LIKE\_RECEPTOR\_SIGNALING\_PATHWAY |  | 64 | -0.25 | -1.00 | 0.443 | 0.763 | 1.000 | 3279 | tags=16%, list=11%, signal=17% |
| 37 | KEGG\_BLADDER\_CANCER |  | 39 | -0.28 | -0.98 | 0.468 | 0.804 | 1.000 | 4348 | tags=26%, list=14%, signal=30% |
| 38 | KEGG\_RNA\_POLYMERASE |  | 26 | -0.30 | -0.96 | 0.522 | 0.851 | 1.000 | 3041 | tags=15%, list=10%, signal=17% |
| 39 | KEGG\_SNARE\_INTERACTIONS\_IN\_VESICULAR\_TRANSPORT |  | 38 | -0.27 | -0.96 | 0.512 | 0.832 | 1.000 | 3792 | tags=18%, list=12%, signal=21% |
| 40 | KEGG\_GLIOMA |  | 64 | -0.24 | -0.94 | 0.592 | 0.852 | 1.000 | 4061 | tags=17%, list=13%, signal=20% |
| 41 | KEGG\_GLYCOSAMINOGLYCAN\_DEGRADATION |  | 21 | -0.31 | -0.94 | 0.547 | 0.847 | 1.000 | 426 | tags=10%, list=1%, signal=10% |
| 42 | KEGG\_GLUTATHIONE\_METABOLISM |  | 50 | -0.25 | -0.93 | 0.582 | 0.862 | 1.000 | 9433 | tags=40%, list=30%, signal=57% |
| 43 | KEGG\_TRYPTOPHAN\_METABOLISM |  | 36 | -0.26 | -0.92 | 0.592 | 0.856 | 1.000 | 6441 | tags=36%, list=21%, signal=46% |
| 44 | KEGG\_PPAR\_SIGNALING\_PATHWAY |  | 67 | -0.22 | -0.90 | 0.657 | 0.896 | 1.000 | 2470 | tags=13%, list=8%, signal=15% |
| 45 | KEGG\_TOLL\_LIKE\_RECEPTOR\_SIGNALING\_PATHWAY |  | 98 | -0.20 | -0.87 | 0.747 | 0.960 | 1.000 | 6422 | tags=26%, list=21%, signal=32% |
| 46 | KEGG\_HISTIDINE\_METABOLISM |  | 27 | -0.26 | -0.87 | 0.661 | 0.942 | 1.000 | 9366 | tags=44%, list=30%, signal=64% |
| 47 | KEGG\_GLYCOLYSIS\_GLUCONEOGENESIS |  | 59 | -0.22 | -0.86 | 0.724 | 0.931 | 1.000 | 8679 | tags=34%, list=28%, signal=47% |
| 48 | KEGG\_CARDIAC\_MUSCLE\_CONTRACTION |  | 68 | -0.21 | -0.85 | 0.748 | 0.934 | 1.000 | 5515 | tags=21%, list=18%, signal=25% |
| 49 | KEGG\_ONE\_CARBON\_POOL\_BY\_FOLATE |  | 17 | -0.30 | -0.85 | 0.661 | 0.916 | 1.000 | 3096 | tags=12%, list=10%, signal=13% |
| 50 | KEGG\_PROTEASOME |  | 42 | -0.23 | -0.83 | 0.769 | 0.942 | 1.000 | 12363 | tags=52%, list=40%, signal=87% |
| 51 | KEGG\_ARGININE\_AND\_PROLINE\_METABOLISM |  | 51 | -0.22 | -0.83 | 0.773 | 0.925 | 1.000 | 4483 | tags=18%, list=14%, signal=21% |
| 52 | KEGG\_SPLICEOSOME |  | 107 | -0.19 | -0.83 | 0.839 | 0.921 | 1.000 | 7351 | tags=23%, list=24%, signal=31% |
| 53 | KEGG\_ECM\_RECEPTOR\_INTERACTION |  | 82 | -0.20 | -0.82 | 0.838 | 0.923 | 1.000 | 3194 | tags=13%, list=10%, signal=15% |
| 54 | KEGG\_GLYOXYLATE\_AND\_DICARBOXYLATE\_METABOLISM |  | 16 | -0.28 | -0.81 | 0.739 | 0.916 | 1.000 | 2682 | tags=13%, list=9%, signal=14% |
| 55 | KEGG\_DRUG\_METABOLISM\_CYTOCHROME\_P450 |  | 71 | -0.20 | -0.80 | 0.850 | 0.927 | 1.000 | 8787 | tags=31%, list=28%, signal=43% |
| 56 | KEGG\_REGULATION\_OF\_AUTOPHAGY |  | 33 | -0.23 | -0.77 | 0.836 | 0.941 | 1.000 | 3279 | tags=15%, list=11%, signal=17% |
| 57 | KEGG\_NUCLEOTIDE\_EXCISION\_REPAIR |  | 42 | -0.20 | -0.72 | 0.920 | 0.986 | 1.000 | 8632 | tags=36%, list=28%, signal=49% |
| 58 | KEGG\_PARKINSONS\_DISEASE |  | 92 | -0.17 | -0.72 | 0.960 | 0.973 | 1.000 | 10400 | tags=34%, list=34%, signal=51% |
| 59 | KEGG\_RENIN\_ANGIOTENSIN\_SYSTEM |  | 17 | -0.24 | -0.69 | 0.880 | 0.981 | 1.000 | 10254 | tags=41%, list=33%, signal=62% |
| 60 | KEGG\_ABC\_TRANSPORTERS |  | 44 | -0.18 | -0.67 | 0.959 | 0.977 | 1.000 | 4338 | tags=16%, list=14%, signal=18% |
| 61 | KEGG\_ETHER\_LIPID\_METABOLISM |  | 29 | -0.15 | -0.50 | 0.996 | 0.998 | 1.000 | 6894 | tags=24%, list=22%, signal=31% |
Table: Gene sets enriched in phenotype **W (5 samples)**[plain text format]****

  
