# Supplementary material for: Mutation analysis of the GSDME gene in a Chinese family with non-syndromic hearing loss
Source: PLoS One. 2022 Nov 9;17(11):e0276233. doi: 10.1371/journal.pone.0276233 (PMC9645625; doi:10.1371/journal.pone.0276233)
Supplement: S3 File — (ZIP) [file pone.0276233.s005.zip › S3_File/Gsea/index.html]

Index for xtools.gsea.Gsea 2.Gsea.1659660506975

### GSEA Report for Dataset biaoda

#### Enrichment in phenotype: **K (6 samples)**

- 114 / 175 gene sets are upregulated in phenotype **K**- 4 gene sets are significant at FDR < 25%- 10 gene sets are significantly enriched at nominal pvalue < 1%- 18 gene sets are significantly enriched at nominal pvalue < 5%- Snapshot of enrichment results- Detailed enrichment results in html format- Detailed enrichment results in TSV format (tab delimited text)- Guide to interpret results

#### Enrichment in phenotype: **W (5 samples)**

- 61 / 175 gene sets are upregulated in phenotype **W**- 15 gene sets are significantly enriched at FDR < 25%- 4 gene sets are significantly enriched at nominal pvalue < 1%- 11 gene sets are significantly enriched at nominal pvalue < 5%- Snapshot of enrichment results- Detailed enrichment results in html format- Detailed enrichment results in TSV format (tab delimited text)- Guide to interpret results

#### Dataset details

- The dataset has 30994 features (genes)- No probe set => gene symbol collapsing was requested, so all 30994 features were used

#### Gene set details

- Gene set size filters (min=15, max=500) resulted in filtering out 11 / 186 gene sets- The remaining 175 gene sets were used in the analysis- List of gene sets used and their sizes (restricted to features in the specified dataset)

#### Gene markers for the **K** *versus* **W** comparison

- The dataset has 30994 features (genes)- # of markers for phenotype **K**: 15325 (49.4% ) with correlation area 50.1%- # of markers for phenotype **W**: 15669 (50.6% ) with correlation area 49.9%- Detailed rank ordered gene list for all features in the dataset- Heat map and gene list correlation  profile for all features in the dataset

#### Global statistics and plots

- Plot of p-values *vs.* NES- Global ES histogram

#### Other

- Parameters used for this analysis

#### Comments

- Timestamp used as random seed: 1659660506975

#### Citing GSEA and MSigDB

To cite your use of the GSEA software please reference the following:

- Subramanian, A., Tamayo, P., et al. (2005, PNAS). - Mootha, V. K., Lindgren, C. M., et al. (2003, Nature Genetics).

For use of the Molecular Signatures Database (MSigDB), to cite please reference   
one or more of the following as appropriate, along with the source for the gene set as listed on the gene set page:

- Liberzon A, et al. (Bioinformatics, 2011). - Liberzon A, et al. (Cell Systems 2015).

---

Report: 2.Gsea.1659660506975.rpt   by user: of

xtools.gsea.Gsea [Fri, Aug 5, '22 8 AM 48]

Website: www.gsea-msigdb.org/gsea
Questions & Suggestions: Contact page
